# Comparative genome analysis of an avirulent and two virulent strains of avian *Pasteurella multocida* reveals candidate genes involved in fitness and pathogenicity

**DOI:** 10.1186/1471-2180-13-106

**Published:** 2013-05-14

**Authors:** Timothy J Johnson, Juan E Abrahante, Samuel S Hunter, Melissa Hauglund, Fred M Tatum, Samuel K Maheswaran, Robert E Briggs

**Affiliations:** 1 Department of Veterinary and Biomedical Sciences, University of Minnesota, St. Paul, Minnesota, USA; 2 Institute for Bioinformatics and Evolutionary Studies, University of Idaho, Moscow, Idaho, USA; 3 National Animal Disease Center, Agricultural Research Service, US Department of Agriculture, Ames, Iowa, USA

**Keywords:** Pasteurella multocida, Genomics, Genome, Virulence, Avian, Fitness, Fowl cholera, Pathogenicity

## Abstract

**Background:**

*Pasteurella multocida* is the etiologic agent of fowl cholera, a highly contagious and severe disease of poultry causing significant mortality and morbidity throughout the world. All types of poultry are susceptible to fowl cholera. Turkeys are most susceptible to the peracute/acute forms of the disease while chickens are most susceptible to the acute and chronic forms of the disease. The whole genome of the Pm70 strain of *P. multocida* was sequenced and annotated in 2001. The Pm70 strain is not virulent to chickens and turkeys. In contrast, strains X73 and P1059 are highly virulent to turkeys, chickens, and other poultry species. In this study, we sequenced the genomes of *P. multocida* strains X73 and P1059 and undertook a detailed comparative genome analysis with the avirulent Pm70 strain. The goal of this study was to identify candidate genes in the virulent strains that may be involved in pathogenicity of fowl cholera disease.

**Results:**

Comparison of virulent versus avirulent avian *P. multocida* genomes revealed 336 unique genes among the P1059 and/or X73 genomes compared to strain Pm70. Genes of interest within this subset included those encoding an L-fucose transport and utilization system, several novel sugar transport systems, and several novel hemagglutinins including one designated PfhB4. Additionally, substantial amino acid variation was observed in many core outer membrane proteins and single nucleotide polymorphism analysis confirmed a higher dN/dS ratio within proteins localized to the outer membrane.

**Conclusions:**

Comparative analyses of highly virulent versus avirulent avian *P. multocida* identified a number of genomic differences that may shed light on the ability of highly virulent strains to cause disease in the avian host, including those that could be associated with enhanced virulence or fitness.

## Background

Avian pasteurellosis, also known as fowl cholera is a highly contagious, systemic, and severe disease affecting wild and domestic birds frequently resulting in high mortality and morbidity. The disease is of major economic importance throughout the world in areas of domestic poultry production [1-3]. The causative agent of fowl cholera is *Pasteurella multocida,* a Gram-negative bacterium. Carter [4,5] identified five capsular types of *P. multocida* based on differences in capsular antigens and designated them as A, B, D, E, and F serogroups. Heddleston and co-workers classified the bacterium into 16 somatic types based on differences in the lipopolysaccharide antigens [6]. In 1981, a standard system for identifying serotypes of *P. multocida* was recommended that combined both the Carter capsular typing and Heddleston somatic typing systems to designate serotypes [7] and a serotype is designated by its capsular type followed by the somatic type. Using this system, the most common serotypes causing fowl cholera in the United States are A:1, A:3, and A:3.4 [8]. While there are no indications that any particular serotype is more or less virulent than others the virulence of avian isolates of most common serotypes appears to vary considerably [9].

Fowl cholera disease can occur in peracute/acute and subacute/chronic forms [10]. All types of poultry are susceptible to the disease, although among them turkeys, pheasants and partridges are highly susceptible to peracute/acute forms of disease whereas chickens are relatively more resistant [11]. In chickens, the most common forms of the disease are acute and chronic. In peracute/acute disease there is sudden death due to terminal – stage bacteremia and endotoxic shock [1,3]. Signs of acute cholera have been reproduced by injection of endotoxin from *P. multocida*[12-14]. Post-mortem findings are dominated by general septicemic lesions. [1,2]. In chronic disease, signs are principally due to localized infections of leg or wing joints, comb, wattles and subcutaneous tissue of the head [2,10]. The completed genome of *P. multocida* strain Pm70 has been available for over eleven years [15] and has greatly facilitated subsequent genomic-based approaches towards better understanding the underlying genetic mechanisms related to virulence and fitness. This complete genome sequence has been used in the study of specific enzymes [16], microarray analyses of differentially expressed genes [17-20], proteomic analyses [21,22], study of virulence factors [16,23-25], reverse vaccinology approaches [26], and as a reference for assembly and comparison to other genomes. While the Pm70 genome sequence has been a great asset in our studies, progress has been modest in the identification and understanding of *P. multocida* virulence [27]. Even today, very little is known about the totality of the mechanisms behind *P. multocida’s* ability to cause disease. The Pm70 strain was isolated from the oviduct of a layer chicken in 1976 from Texas (personal communication- RE. Briggs). This strain belongs to serotype F:3 [28] and not A:3 as reported earlier [15], is avirulent and does not cause experimental fowl cholera disease in chickens [28]. In contrast, other strains of *P. multocida* have been isolated, such as strains X73 and the P1059, that are highly virulent to chickens, turkeys, and other poultry species [29,30].

Additional *P. multocida* strains of bovine, avian, and porcine origin have recently been sequenced, which was the subject of a recent comparative review [31]. The authors noted, based on the nine genomes sequenced to date, there was “no clear correlation between phylogenetic relatedness and host predilection or disease”. Information is sparse on the location and characterization of the genes responsible for differences in virulence of avian and other *P. multocida*. In another recent review, Boyce et al. [32] speculated that the combination of additional *P. multocida* genome sequences and advances in our ability to genetically manipulate the organism will facilitate major advances in our understanding of disease pathogenesis. To that end, we undertook a detailed comparative genome analysis of two virulent strains (X73 and P1059) and avirulent strain Pm70 of *P. multocida*. The goal of this study is to enable narrowed identification of a repertoire of unique genes present in the highly pathogenic avian strains that may play a role in virulence. This information will also facilitate the design of improved modified live vaccine candidates with defined mutations that can be evaluated as immunoprophylactic agent(s) to control *P. multocida*-caused disease in avian and other host species.

## Methods

### Bacterial strains

The strains sequenced in this study included *P. multocida* strains P1059 (ATCC# 15742) and X73 (ATCC# 11039). Strain P1059 is a well characterized pathogenic strain isolated from the liver of a turkey that died of fowl cholera [30]. Strain X73 is also a well characterized pathogenic strain isolated from the liver of a chicken that died of fowl cholera [30]. For comparative purposes, the avirulent Pm70 strain was used [15]. There are several reasons why we selected strains P1059 and X73 in this study. First, both strains are highly virulent to chickens, turkeys and other poultry species. Second, they are of different serotypes (P1059 = A:3; and X73 = A:1) and different immunologic types [30]. Thirdly, they are reference serotype strains that are readily available to investigators and there is abundant literature on the biology of these two strains [1,11,30,33-35].

### Genome sequencing and annotation

Sequencing of strains P1059 and X73 was performed using 454 Life Sciences pyrosequencing at the National Animal Disease Center in Ames, Iowa. The following data sets were generated for each strain: GS- FLX, with 270,010 shotgun reads of average length 240 bp yielding 64,827,159 bp for P1059; and GS-FLX, with 227,030 shotgun reads of average length of 240 bp, yielding 54,398,540 bp for X73. Reads were *de novo* assembled into scaffolds using Newbler 2.3 [36]. The draft sequences of these genomes are deposited under the following accession numbers: P1059 [Genbank:AMBQ01000000] and X73 [Genbank:AMBP01000000].

### Comparative genomics

Annotation of P1059 and X73 was performed using publicly available tools. Putative coding regions were predicted using GeneMarkS [37]. Gene function was assigned using HMMER3 against Pfam-A 24.0, RPS-BLASTp against CDD, and BLASTp against all microbial proteins [38,39]. tRNA genes were identified using tRNAscan-SE [40]. rRNA genes were identified using RNAmmer [41]. For analysis of the shared and unique proteins in the *P. multocida* genomes sequenced, BlastP was used with a similarity cutoff of 90% identity over 90% of the protein as an arbitrary designation for similar versus dissimilar proteins. For genomic island analysis, whole genome alignments were performed using MAUVE to identify regions present in strains P1059 and X73 but absent from strain Pm70 [42]. Linear and circular genomic maps were generated using XPlasMap and Circos [43]. Single nucleotide polymorphism (SNP) analysis was performed using SNPeff [44].

## Results and discussion

### Overview of the *P. multocida* P1059 and X73 genomes

A total of 270,010 reads were used to draft assemble strain P1059, resulting in a single scaffold of 27 large contigs (> 500 bp) of approximately 27-fold coverage and an estimated genome size of 2.4 Mb. A total of 227,030 reads were used to draft assemble strain X73, resulting in 17 large contigs (> 500 bp) of approximately 23-fold coverage and an estimated genome size of approximately 2.4 Mb. No plasmids were identified in either strain sequenced. The contigs generated were then aligned to strain Pm70 to generate collinear draft sequences and subsequently compare the three avian source genomes.

### Unique regions of virulent avian *P. multocida*

**Figure 1:**
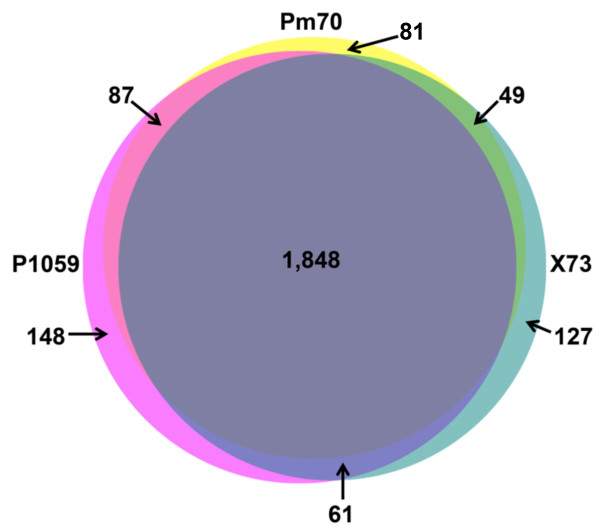
**Venn diagram illustrating the shared and unique proteins of *P. multocida *strains Pm70, P1059, and X73.**

**Figure 2:**
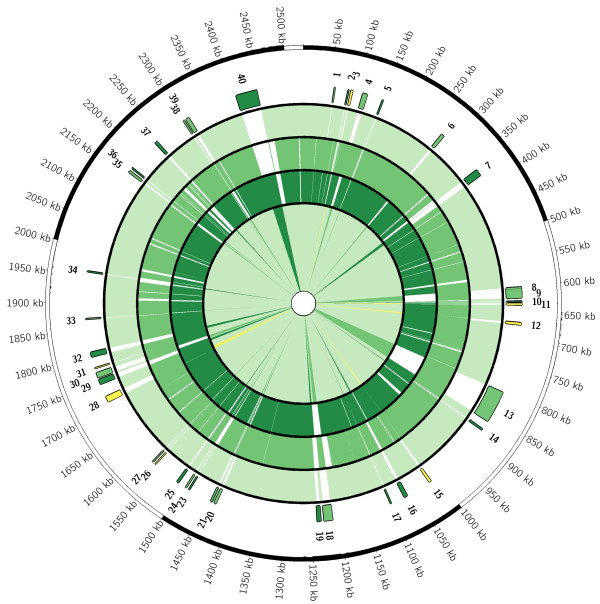
**Circular map comparing sequenced avian source *P. multocida *strains.** Scale is presented in kb. The outermost rings depict genomic regions not present in strain Pm70 but present in strains P1059 (light green), X73 (dark green), or both (yellow). Regions are numbered as described in the Tables. The next three rings depict the shared genomic regions of avian source strains Pm70 (outer ring), P1059 (middle ring), and X73 (inner ring). Colored regions depict regions present at greater than 90% nucleotide similarity and non-colored regions depict regions absent. The innermost ring again depicts the core (very light green) regions present in all three strains and the regions absent from strain Pm70 but present in other sequenced strains using the same color scheme.

The draft genomes of strains P1059 and X73 were found to contain 2,144 and 2,085 predicted proteins, respectively. Along with strain Pm70, the genomes all contained 51 tRNA-carrying genes and 4 rRNA-carrying operons. The genomes of the three avian *P. multocida* strains contained a remarkably high number of shared proteins (1,848), which comprised 86.2-90.7% of the predicted proteins of the three avian strains using a BlastP similarity cut-off of 90% (Figure 1). Compared to strain Pm70, a total of 336 unique proteins were identified in either strains P1059 or X73, of which 61 were contained within both genomes (Table 1). Most of the 61 shared proteins were small predicted proteins of unknown function and located individually throughout the *P. multocida* genome that could be attributed to differences in annotation approaches (Figure 2). Also, most of the predicted proteins identified were present in one or more sequenced *P. multocida* from the NCBI database that were not from avian hosts. However, one noteworthy region of difference shared by P1059 and X73, but absent from Pm70 and other strains of non-avian source, was located between the core genes *deoC* and *rfaD* in both P1059 and X73 (P1059 – 01496 to 01503; X73 – 01400 to 01407). This region contained ten predicted proteins with similarity to systems involved in the transport and utilization of L-fucose. L-fucose is an important component of host mucin and has shown to be a chemoattractant for certain bacterial species, such as *Campylobacter jejuni*. Moreover, the ability to utilize L-fucose by *C. jejuni* has been shown to confer a fitness advantage for avian strains in low nutrient environments such as the respiratory tract [45,46]. Comparison of available *P. multocida* sequences suggests that the presence of this region may be a defining feature of pathogenic avian-source *P. multocida* strains, as it was present in P1059, X73, and *P. multocida* subsp. *gallicida* strain Anand1 isolated from a chicken in India [47] but absent from strains Pm70, pathogenic bovine-source strain 36950 [48] and pathogenic swine source strains 3480 and HN06 [49]. Other studies have demonstrated an ability of avian-source *P. multocida* to ferment L-fucose, further suggesting that the majority of avian-source *P. multocida* strains harbor this system [9,33,50]. Other bacteria inhabiting the respiratory tracts of poultry have been identified to utilize L-fucose, such as *Gallibacterium anatis*, suggesting that such capabilities may be advantageous for respiratory bacterial pathogens of poultry [51]. Such systems could play a role in increased fitness and/or virulence capability of strains P1059 and X73 in the avian host.

**Table 1:** **Predicted proteins of interest present in *P. multocida *strains P1059 and X73 at greater than 90% similarity but absent from strain Pm70**

				**Presence in:**					
**Gene locus (P1059)**	**Length (aa)**	**Genomic island**	**Predicted function**	**Pm70**	**P1059**	**X73**	**36950**	**HN06**	**3480**
00226	66	NA	Hypothetical protein	-	+	+	-	+	+
00545	68	NA	Hypothetical protein	-	+	+	-	+	+
00580	828	12	Trimethylamine-N-oxide reductase	-	+	+	+	+	+
00581	371	12	Cytochrome c-type protein TorY	-	+	+	+	+	+
00881	1125	15	Putative Ton-B dependent heme receptor	-	+	+	-	-	-
00948	62	NA	Hypothetical protein	-	+	+	+	+	+
01347	332	26	Putative DNA-binding protein	-	+	+	+	+	+
01412	52	NA	Hypothetical protein	-	+	+	+	+	+
01496	249	28	L-fucose operon activator	-	+	+	-	-	-
01497	586	28	L-fucose isomerase	-	+	+	-	-	-
01498	495	28	L-fuculokinase	-	+	+	-	-	-
01499	144	28	L-fucose mutarotase	-	+	+	-	-	-
01500	215	28	L-fuculose phosphate aldolase	-	+	+	-	-	-
01501	508	28	Ribose ABC transport system, ATP-binding protein	-	+	+	-	-	-
01502	342	28	Ribose ABC transport system, permease protein	-	+	+	-	-	-
01503	318	28	Ribose ABC transporter, periplasmic ribose-binding protein	-	+	+	-	-	-
01505	480	28	Aldehyde dehydrogenase A	-	+	+	-	-	-
01550	384	31	Flavohemoprotein	-	+	+	+	-	+
01587	53	NA	Hypothetical protein	-	+	+	+	+	+
01686	108	NA	HigA antitoxin protein	-	+	+	-	+	-
01825	60	NA	Hypothetical protein	-	+	+	+	+	+
01854	51	NA	Hypothetical protein	-	+	+	-	-	+
01963	52	NA	Hypothetical protein	-	+	+	+	+	+

Presence of these proteins in additional sequenced *P. multocida* is also presented.

Twelve proteins were also identified that were present in both strains P1059 and X73 at greater than 90% amino acid similarity, but at less than 90% similarity in strain Pm70 (Table 2). Among the twelve proteins identified were several membrane-associated proteins, including LspB, PfhB3, Opa, and SprT. The presence of divergent protein sequences that are membrane-associated is suggestive of adaptation of *P. multocida* strains towards particular hosts.

**Table 2:** **Predicted proteins of interest present in *P. multocida *strains X73 and P1059 at greater than 90% similarity but present at less than 90% similarity in strain Pm70**

**Gene locus**	**Length (aa)**	**Predicted function**
00056	576	Hemolysin activator protein precursor
00060	1767	Exoprotein involved in heme utilization or adhesion - PfhB3
00219	96	Hypothetical protein
00361	617	Outer membrane iron receptor protein-Fe transport
00444	80	Hypothetical protein
00514	116	Hypothetical protein
00515	91	Hypothetical protein
00522	70	Hypothetical protein
00795	972	Beta-1,3-glucosyltransferase
01068	197	Opacity family integral membrane protein-Opa protein
01069	169	SprT- protein
01350	424	Nucleoside permease -NupC

**Figure 3:**
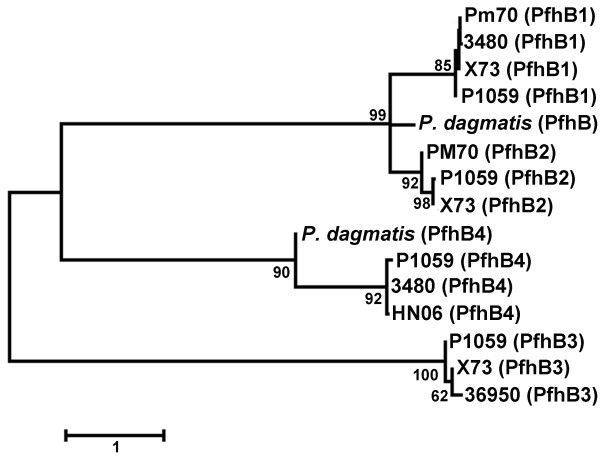
**Dendrogram depicting amino acid sequence similarities between the filamentous heagglutinins of *Pasteurella multocida*.** Evolutionary history was inferred using the Maximum Likelihood method based on the JTT matrix-based model. The tree is drawn to scale, and 500 bootstrap iterations were performed. A total of 1,479 positions were used in the final dataset. The analyses were conducted in MEGA [Tamura et al. 2007]. Proteins from *P. dagmatis* were included for comparative purposes.

There were also predicted proteins identified as unique to strains P1059 (148 total) and X73 (127 total) compared to strain Pm70. Many of these proteins were again of unknown function and/or associated with prophage-like elements (Additional file 1: Table S1 and Additional file 2: Table S2). However, some systems unique to each strain were noteworthy. In strain P1059, one unique region contained six genes predicted as involved in the transport and modification of citrate, and the conversion of citrate to oxaloacetate via citrate lyase (00080 to 00085). This system was absent in all other sequenced *P. multocida* genomes. The conversion of citrate to oxaloacetate is linked to citrate fermentation. Also unique to strain P1059, but present in strains 36950, 3480, and HN06, are four genes involved in xylose ABC transport system with a transcriptional repressor (01538 to 01541). Present in strains X73 and 36950 was a putative toxin-antitoxin system similar to the HipAB systems (genes 02005 and 02006). Finally, genes for several novel proteins with similarity to the previously described Pfh-type filamentous hemagglutinins were identified in strains P1059 and X73. Strain P1059 contained a novel predicted filamentous hemagglutinin (designated PfhB4 – gene # 00523) that shares similarity with PfhB1 and PfhB2 from *P. multocida*. PfhB4 has conserved domains related to hemagglutination activity, two-partner secretion, hemagglutinin repeats, and toxicity. PfhB4 is present only in strains P1059, HN06, and 3480 (Figure 3). It is adjacent to a putative hemolysin secretion/activation protein (gene # 00522) and thus appears to represent a novel two-partner system involved in hemagglutination. PfhB2 of strain P1059 has been shown to play an important role in either colonization or invasion in the turkey model [34]. Also, vaccination with recombinant P1059 PfhB2 peptides cross protected turkeys against an X73 challenge [35]. PfhB2 was present in strain Pm70, P1059, and X73, but was only 90% similar in the latter two as compared to Pm70. Overall, the presence of unique genes/systems related to metabolism and adhesion could provide strains such as P1059 with additional tools for increased fitness leading to higher virulence.

Of the 127 unique proteins identified in strain X73 were five genes for a galactitol-specific phosphotransferase and utilization system (00310 to 00316), only present in strain X73; three genes for a TRAP dicarboxylate transporter system (01441 to 01443), also present in strain 36950; and six genes for a novel simple sugar D-allose transport and utilization systems (00951 to 00956), only present in strain X73. Such systems could again provide additional means of energy production in a resource-limited environment.

### Known virulence factors and antigens

Comparisons were performed for several known virulence factors and outer membrane proteins that are important for *P. multocida* pathogenesis, functionality, and vaccine development [52]. These comparisons revealed some noteworthy aspects relative to their presence and evolution in *P. multocida*. For example, the hemoglobin receptors *hgbA* and *hgbB* were present in all sequenced *P. multocida* genomes, but are significantly different in their amino acid similarities (Table 3). HgbA and HgbB have been shown to exhibit hemoglobin binding properties [53,54]. Their incomplete distribution reported in previous studies could be attributed to genetic variation rather than complete absence of these genes [55]. The outer membrane porins *ompH1* and *ompH2* were also present in all sequenced strains, with *ompH2* more highly conserved than *ompH1* with respect to amino acid similarity. Furthermore, a third outer membrane porin *ompH3* was present in all sequenced strains except strain X73, but was highly conserved within these strains. The *ptfA* gene, encoding a type 4 fimbrial subunit, was highly conserved in all sequenced strains, as was *comE* encoding a fibronectin-binding protein. The *pfhB1* gene, encoding a filamentous hemagglutinin protein, was present in strains Pm70, P1059, X73, and 3480. PfhB1 was highly conserved among these strains. PfhB2, a second filamentous hemagglutinin, was present in strains Pm70, P1059, X73, and 36950. This protein was more variable in amino acid sequence among these strains (Figure 3). Two other genes encoding filamentous hemaggultinins, *pfhB3* and *pfhB4*, were absent in strain Pm70, with *pfhB3* present in strains P1059, X73, and 36950, and *pfhB4* present in strains P1059, HN06, and 3480. Finally, lipoproteins *plpP*, *plpB*, and *plpD* were present in all sequenced strains, and all were highly conserved except *plpP*, whose product shared only 82-98% amino acid similarity between strains.

**Table 3:** **Similarity of proteins of interest in sequenced avian *Pasteurella multocida *genomes**

**Protein name**	**Pm70**	**P1059**	**X73**	**36950**	**HN06**	**3480**
HgbA	100^A^	87	96	89	99	99
HgbB	100	-	95	-	84	-
Omp16	100	100	100	99	100	100
OmpH1	100	84	83	83	84	99
OmpH2	100	98	98	99	98	97
OmpH3	100	97	-	98	97	98
TbpA	100	99	99	98	100	99
PtfA	100	100	100	100	100	99
ComE	100	99	100	99	99	99
PlpE	100	94	94	-	-	-
PlpP	100	84	82	98	72	76
PlpB	100	99	100	99	100	100
PlpD	100	100	100	100	100	100
PfhB1 (PM0057)	100	99	98	-	-	99
PfhB2 (PM0059)	100	90	90	97	-	-
PfhB3	-	100^B^	98	96	-	-
PfhB4	-	100	-	-	93	93

^A^Percent amino acid similarity to same protein from strain Pm70.

^B^Percent amino acid similarity to same protein from strain P1059.

### Single nucleotide polymorphisms

**Figure 4:**
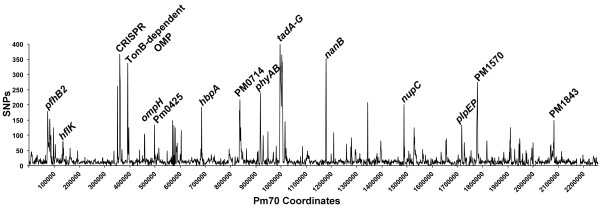
**Density map of single nucleotide polymorphisms (SNPs) between strains Pm70, P1059, and X73 across the *Pasteurella multocida *strain Pm70 genome conserved in all strains.** SNPs were identified using MAUVE and included genomic regions present in all three strains.

The three avian source *P. multocida* genomes were also compared for SNPs within the conserved regions of their genomes using MAUVE [42], and the SNPs were analyzed for their coding effects using SNPeff [44] (Table 4). A total of 31,021 SNPs were identified between strains Pm70 and P1059, and 26,705 SNPs were identified between strains Pm70 and X73. The density of SNPs varied considerably across the *P. multocida* genome, with some regions containing a much higher density of SNPs than the rest of the core genome (Figure 4). This suggests that some regions of the genome are under diversifying selection, while the majority of the genome is under neutral or purifying selection. The ratio between non-synonymous to synonymous substitutions (dN/dS) is commonly used as a measure of purifying versus diversifying selection [56]. The overall dN/dS ratios of all coding regions of strains P1059 and X73 compared to strain Pm70 were 0.40 and 0.38, respectively. Proteins were then divided into groups based upon predicted subcellular localization of each protein using PSORT-B version 3.0. Using this approach, the dN/dS ratios varied considerably, with higher ratios (0.76-0.93) found within proteins predicted as extracellular or outer membrane [57]. Amongst specific outer membrane proteins, the highest dN/dS ratios were observed within PfhB2, HgbA, HemR, pm0591 (a secreted effector protein), pm0803 (an iron-regulated outer membrane protein), TadD-F (pilus assembly proteins), RcpB-C (pilus assembly proteins), and PlpP. The higher dN/dS ratios observed among this subset of extracellular and outer membrane proteins is suggestive that they are under diversifying selection due to interactions with the host immune system, although further analyses would be required to confirm this observation.

**Table 4:** **Single nucleotide polymorphism (SNP) analysis and dN/dS ratios of categorized and selected coding regions of *Pasteurella multocida *strains Pm70, P1059, and X73**

	**Location**	**Non-synonymous**	**Synonymous**	**dN/dS**
Pm70 vs. P1059	Total	8910	22111	0.4
	Cytoplasmic	2431	9933	0.25
	Cytoplasmic membrane	1556	5556	0.28
	Extracellular	94	103	0.91
	Outer membrane	1575	2062	0.76
	Periplasmic	93	549	0.17
Pm70 vs. X73	Total	7401	19304	0.38
	Cytoplasmic	2384	9162	0.26
	Cytoplasmic membrane	1251	4710	0.27
	Extracellular	125	134	0.93
	Outer membrane	1783	1976	0.9
	Periplasmic	98	593	0.17
	**Function**	**Non-synonymous**	**Synonymous**	**dN/dS**
PfhR (pm0040)	Putative porin-Fe transport	7	15	0.47
PfhB1 (pm0057)	Filamentous hemagglutinin	34	65	0.52
PfhB2 (pm0059)	Filamentous hemagglutinin	498	506	0.98
Est (pm0076)	Outer membrane esterase	39	59	0.66
PtfA (pm0084)	Type IV fimbrial subunit-ptfA	4	0	4
HgbA (pm0300)	TonB-dependent hemoglobin receptor	159	152	1.05
Csy1 (pm0305)	CRISPR-associated protein	290	130	2.23
OmpW (pm0331)	Outer membrane protein	2	4	0.5
pm0336	TonB-dependent receptor	39	57	0.68
HgbB (pm0337)	Hemoglobin binding protein	78	90	0.87
OmpH_1 (pm0388)	Outer membrane porin	36	66	0.55
OmpH_2 (pm0339)	Outer membrane porin	10	16	0.63
TolC1 (pm0527)	Outer membrane efflux channel	12	44	0.27
Pcp (pm0554)	Peptidoglycan-associated protein	0	3	0
HemR (pm0576)	Hemoglobin binding receptor	6	4	1.5
pm0591	Secreted effector protein	75	40	1.88
PhyA (pm0773)	Capular polysacharride export protein	2	4	0.5
OmpA (pm0786)	Outer membrane protein	61	70	0.87
Pm0803	Outer membrane receptor protein, mostly Fe transport	67	58	1.16
TadF (pm0844)	Pilus assembly protein	112	81	1.38
TadE (pm0845)	Pilus assembly protein	134	70	1.91
TadD (pm0846)	Pilus assembly protein	126	103	1.22
RcpB (pm0851)	Pilus assembly protein	144	69	2.08
RcpA (pm0852)	Pilus assembly protein	182	222	0.82
RcpC (pm0853)	Pilus assembly protein	166	112	1.48
Flp1 (pm0855)	Flp pilin component	21	19	1.11
pm0998	Hypothetical protein	6	4	1.5
NanB (pm1000)	Outer membrane sialydase	157	161	0.98
TonB (pm1188)	TonB energy supply via iron transport	3	4	0.75
GlpQ (pm1444)	Glycerophosphodiester	2	3	0.67
PlpE (pm1517)	Protective outer membrane lipoprotein	24	39	0.62
PlpP (pm1518)	Protective outer membrane lipoprotein	63	55	1.13
TorD (pm1794)	Chaperone	4	3	1.33

### LPS genes

The Heddleston somatic typing system classifies *P. multocida* into 16 somatic types based on antigenic differences in the lipopolysaccharide (LPS) [6]. Good progress has been made in understanding the structural basis for the LPS typing scheme. The genes and the transferases required for the biosynthesis of the somatic type-specific outer core region of the LPS has been identified in strains of *P. multocida* strains representing various somatic types [24,58-63]. Since endotoxin (LPS) is a key virulence factor in *P. multocida*, we examined each gene involved in LPS biosynthesis in the X73 and P1059 strains and compared with the Pm70 strain. All three strains produced two glycoforms simultaneously, termed glycoforms A and B. Both X73 and P1059 contained the inner core biosynthetic complement of genes, including *kdtA* (P1059-01455; X73- 01363), *hptA* (*opsX*; P1059-02017; X73- 01921), *kdkA* (P1059-01451; X73-01359), *hptC* (*rfaF;* P1059-02018 ; X73-01922), *hptD* (P1059-01443; X73-01351 ) and *gctA* (P1059-01456; X73-01364). The gene that encodes for the enzyme which catalyzes the attachment of phosphoethanolamine to L-α-D Heptose −11 (Pm70-pm0223) was present only in strains P1059 and Pm70. There appeared to be some variation in the *hptD* gene between Pm70 and the X73 and P1059 strains although it was generally conserved between strains. Linking the inner core to the outer core is the *hptE* gene, present in both X73 and P1059 (X73-01185; P1059-01293). The outer core structure expressed by X73, P1059 and Pm70 strains are structurally distinct and distal part of the molecule because in all three strains a polymeric O antigen was absent. The X73 strain but not P1059 and Pm70 express an outer core oligosaccharide that contains two terminal galactose residues, with phosphocholine (PCho). Present in X73 but absent from Pm70 and P1059 were the outer core biosynthetic genes involved in phosphocholine (PCho) biosynthesis genes for somatic type 1. As reported previously [23], these genes include *pcgA* (X73-01180), *pcgB* (X73-01182), *pcgC* (X73-01181), and *pcgD* (X73-01183) as well as *gatA* (X73-01184). X73 attaches a phosphoethanolamine (PEtn) residue to the terminal galactose. Studies have shown [23] that PCho on the LPS is important for virulence of X73 strain to chickens. However, a clear role for PEtn has not been defined. Present in the outer core of Pm70 and P1059, but absent in X73, were the biosynthetic genes for somatic type 3. These genes include *losA* (Pm70-Pm1143; P1059-01292); (Pm70-Pm1138; P1059-01287); (Pm70-Pm1139; P1059-01288); (Pm70-Pm1140; P1059-01289); and (Pm70- Pm1141; P1059-01290).

In summary, comparative analyses of highly virulent versus avirulent *P. multocida* identified a number of genomic differences that may shed light on the ability of highly virulent strains to cause disease in the avian host. Most of the differences observed involved the presence of additional systems in virulent avian-source strains P1059 and/or X73 that appear to play metabolic roles. Such systems might enhance the fitness of these strains in the avian extraintestinal compartment, but without experimental evidence this is purely a speculative observation. This work does, however, underscore the need to utilize such genomic data towards targeted molecular approaches to better understand the role of horizontal gene transfer in the pathogenesis of this organism. Also, it is evident given the high degree of large sequence and single nucleotide polymorphisms in *P. multocida* that focused studies need to be conducted to appreciate adaptation of these strains to their respective hosts.

## Competing interests

The authors declare that they have no competing interests.

## Authors’ contributions

TJJ performed the genomic analysis, and was the primary author of this study. JEA participated in bioinformatics analyses, including sequence annotation, alignments and pathway reconstruction. SSH formatted and prepared assemblies and annotations for submission to GenBank. MH was involved in analyzing the genome sequences. FMT participated in the editorial review of the manuscript. SKM coordinated this study and helped to draft the manuscript. REB conceived this study, performed the genome sequences data and participated in writing of the manuscript. All authors read and approved the final manuscript.

## Supplementary Material

Additional file 1: Table S1 Coding regions present in *Pasteurella multocida* strain P1059 but absent from strains Pm70 and X73, excluding prophage-associated regions. Click here for file

Additional file 2: Table S2 Coding regions present in *Pasteurella multocida* strain X73 but absent from strains Pm70 and P1059, excluding prophage-associated regions. Click here for file
