# Supplementary material for: Comparative genome analysis of an avirulent and two virulent strains of avian Pasteurella multocida reveals candidate genes involved in fitness and pathogenicity
Source: BMC Microbiol. 2013 May 14;13:106. doi: 10.1186/1471-2180-13-106 (PMC3660278; doi:10.1186/1471-2180-13-106)
Supplement: Additional file 1: Table S1 — Coding regions present in Pasteurella multocida strain P1059 but absent from strains Pm70 and X73, excluding prophage-associated regions. [file 1471-2180-13-106-S1.pdf]

Supplementary Table 1. Coding regions present in *Pasteurella multocida* strain P1059 but absent from strains Pm70 and X73, excluding prophage-associated regions. Also shown is the presence of these proteins in other fully sequenced genomes.

Gene locus	Length (aa)	Genomic island	Predicted Function	Pm70	P1059	X73	36950	HN06	3480
00079	45	4	Hypothetical protein	-	+	-	-	-	-
00080	363	4	Citrate lyase ligase	-	+	-	-	-	-
00081	96	4	Citrate lyase gamma chain	-	+	-	-	-	-
00082	291	4	Citrate lyase beta chain	-	+	-	-	-	-
00083	503	4	Citrate lyase alpha chain	-	+	-	-	-	-
00084	482	4	Triphosphoribosyl-dephosphocoenzyme-A synthase	-	+	-	-	-	-
00085	479	4	Citrate carrier/transporter	-	+	-	-	-	-
00086	90	4	Hypothetical protein	-	+	-	+	-	-
00087	38	4	Hypothetical protein	-	+	-	+	-	-
00253	92	6	Hypothetical protein	-	+	-	+	+	-
00254	45	6	Hypothetical protein	-	+	-	+	+	-
00255	39	6	Hypothetical protein	-	+	-	-	-	-
00256	198	6	Hypothetical protein	-	+	-	-	-	-
00257	519	6	Hypothetical protein	-	+	-	-	-	-
00258	571	6	Putative RTX toxins and related calcium binding protein	-	+	-	-	-	-
00333	401	NA	Hypothetical protein	-	+	-	-	-	-
00517	69	8	Hypothetical protein	-	+	-	-	-	-
00522	618	9	Hemolysin activator protein precursor	-	+	-	-	+	+
00523	2868	9	Novel filamentous hemagglutinin PfhB4	-	+	-	-	+	+
00530	187	9	Hypothetical protein	-	+	-	-	-	-
00531	410	9	Putative heme utilization/adhesion protein	-	+	-	-	-	-

00533	52	9	Hypothetical protein	-	+	-	-	-	-
00541	192	NA	Hypothetical protein	-	+	-	-	-	-
00586	154	NA	Hypothetical protein	-	+	-	-	-	-
00895	58	NA	Hypothetical protein	-	+	-	-	-	-
00901	53	NA	Hypothetical protein	-	+	-	-	-	-
00980	167	NA	RcpB protein	-	+	-	-	+	-
01229	71	20	Mobilization protein A, truncated protein	-	+	-	-	-	-
01230	298	20	Cobalt-zinc-cadmium resistant protein	-	+	-	-	-	-
01231	110	20	Plem2 protein	-	+	-	-	-	-
01232	61	20	Hypothetical protein	-	+	-	-	-	-
01235	575	22	Hypothetical protein	-	+	-	-	-	-
01276	331	24	YadA -terminal domain protein	-	+	-	-	-	-
01350	154	NA	Hypothetical protein	-	+	-	-	-	-
01354	217	27	Hypothetical protein	-	+	-	-	-	-
01355	214	27	Hypothetical protein	-	+	-	-	-	-
01462	84	NA	Hypothetical protein	-	+	-	-	-	-
01463	204	NA	Hypothetical protein	-	+	-	-	-	-
01535	440	30	Xylose isomerase	-	+	-	+	+	-
01536	396	30	Aspartate aminotransferase	-	+	-	+	+	+
01537	467	30	Na+/H+ antiporter NhaC	-	+	-	+	+	+
01538	332	30	Xylose ABC transporter, periplasmic xylose-binding protein	-	+	-	+	+	+
01539	504	30	Xylose transport ATP-binding protein	-	+	-	+	+	+
01540	375	30	Ribose ABC transport system, permease protein	-	+	-	+	+	+
01541	389	30	Xylose activator XylR (AraC family)	-	+	-	+	+	+
01905	55	NA	Hypothetical protein	-	+	-	+	-	-
01942	126	NA	Hypothetical protein	-	+	-	-	-	-

01943	188	NA	Hypothetical protein	-	+	-	-	-	-
01948	40	NA	Hypothetical protein	-	+	-	-	-	-
01949	138	NA	Hypothetical protein	-	+	-	-	-	-
02005	435	38	Putative HipA-like toxin	-	+	-	+	-	-
02006	111	38	Putative HipB-like antitoxin	-	+	-	+	-	-
02011	1681	39	Putative helicase	-	+	-	-	-	-
