# Supplementary material for: Comparative genome analysis of an avirulent and two virulent strains of avian Pasteurella multocida reveals candidate genes involved in fitness and pathogenicity
Source: BMC Microbiol. 2013 May 14;13:106. doi: 10.1186/1471-2180-13-106 (PMC3660278; doi:10.1186/1471-2180-13-106)
Supplement: Additional file 2: Table S2 — Coding regions present in Pasteurella multocida strain X73 but absent from strains Pm70 and P1059, excluding prophage-associated regions. [file 1471-2180-13-106-S2.pdf]

Supplementary Table 2. Coding regions present in *Pasteurella multocida* strain X73 but absent from strains Pm70 and P1059, excluding prophage-associated regions. Also shown is protein presence in other sequenced genomes.

Gene locus	Length (aa)	Genomic island	Predicted Function	Pm70	P1059	X73	36950	HN06	3480
00113	317	5	Hypothetical protein	-	-	+	-	-	-
00177	51	NA	Hypothetical protein	-	-	+	-	-	-
00310	317	7	Tagatose 1,6-bisphosphate aldolase	-	-	+	-	-	-
00311	430	7	Tagatose-6-phosphate kinase	-	-	+	-	-	-
00312	151	7	PTS[A] system, galactitol-specific IIA component	-	-	+	-	-	-
00313	94	7	PTS system, galactitol-specific IIB component	-	-	+	-	-	-
00314	451	7	PTS system, galactitol-specific IIC component	-	-	+	-	-	-
00315	347	7	Galactitol-1-phosphate 5-dehydrogenase	-	-	+	-	-	-
00316	252	7	Galactitol utilization operon repressor	-	-	+	-	-	-
00317	129	7	Hypothetical protein	-	-	+	-	-	-
00318	73	7	Hypothetical protein	-	-	+	-	-	-
00319	56	7	Hypothetical protein	-	-	+	-	-	-
00320	271	7	Glycosyltransferase	-	-	+	-	-	-
00321	68	7	Glycosyltransferase	-	-	+	-	-	-
00322	204	7	Hypothetical protein	-	-	+	-	-	-
00323	364	7	UDP-glucose dehydrogenase	-	-	+	-	-	-
00518	82	10	Hypothetical protein	-	-	+	-	+	+
00519	53	10	Hypothetical protein	-	-	+	-	+	+
00773	133	NA	Protein containing domains from glyoxalase/bleomycin resistance	-	-	+	-	-	-
00812	333	16	Transcriptional regulator –Lac1 family	-	-	+	+	-	-
00813	339	16	L-idonate 5-dehydrogenase protein	-	-	+	+	-	-

00814	255	16	5-keto-D-gluconate 5-reductase protein	-	-	+	+	-	-
00815	337	16	TRAP[B] dicarboxylate transporter protein	-	-	+	+	-	-
00816	441	16	Putative gluconate TRAP family transportor protein	-	-	+	+	-	-
00838	526	17	Hypothetical protein	-	-	+	-	-	-
00950	148	19	Ribose-5-phosphate isomerase B	-	-	+	-	-	-
00951	299	19	D-allose kinase protein	-	-	+	-	-	-
00952	222	19	D-allulose-6-phosphate3-epimerase	-	-	+	-	-	-
00953	315	19	D-allose ABC transporter permease component	-	-	+	-	-	-
00954	507	19	D-allose ABC transporter, ATPase component	-	-	+	-	-	-
00955	312	19	D-allose ABC transporter, substrate-binding component	-	-	+	-	-	-
00956	284	19	Transcriptional regulator of D-allose utilization –RpiR family	-	-	+	-	-	-
01120	46	NA	Hypothetical protein	-	-	+	-	-	-
01128	263	21	Hypothetical protein	-	-	+	-	-	-
01164	63	23	Hypothetical protein	-	-	+	-	-	-
01165	63	23	Hypothetical protein	-	-	+	-	-	-
01180	252	25	Hypothetical protein	-	-	+	-	-	-
01181	293	25	Choline kinase	-	-	+	-	-	-
01182	302	25	Choline permease	-	-	+	-	-	-
01183	230	25	Cholinephosphate cytidylyltransferase	-	-	+	-	-	-
01439	394	29	Mannonate dehydratase	-	-	+	+	-	+
01440	248	29	Hexuronate utilization operon transcriptional repressor xUR protein	-	-	+	+	-	+
01441	324	29	TRAP-type4-dicarboxylate transport system, periplasmic component	-	-	+	+	-	+
01442	162	29	TRAP-type transport system,small permease component	-	-	+	+	-	+

01443	436	29	TRAP-type4-dicarboxylate transport system, large permease component	-	-	+	+	-	+
01444	281	29	D-mannonate oxidoreductase	-	-	+	+	-	+
01445	467	29	Uronate isomerase	-	-	+	+	-	+
01446	313	29	2-dehydro-3-deoxygluconate kinase	-	-	+	+	-	+
01484	285	32	Arabinose operon regulatory protein	-	-	+	-	-	-
01485	324	32	Sugar-binding protein	-	-	+	-	-	-
01486	496	32	ABC-type sugar transport system, ATP-binding protein	-	-	+	-	-	-
01487	319	32	Ribose ABC transport system, permease protein RbsC	-	-	+	-	-	-
01488	315	32	Ribose/xylose/arabinose/galactoside ABC-type transport system, permease protein	-	-	+	-	-	-
01489	499	32	Ribulokinase	-	-	+	-	-	-
01490	495	32	L-arabinose isomerase	-	-	+	-	-	-
01607	41	NA	Hypothetical protein	-	-	+	-	-	-
01628	461	34	Type I restriction-modification protein	-	-	+	-	-	-
01629	396	34	Hypothetical protein	-	-	+	-	-	-
01693	263	NA	Sodium channel protein	-	-	+	-	-	-
01824	647	36	Putative phosphatase protein	-	-	+	-	-	-
01856	378	37	Hypothetical protein	-	-	+	-	-	-
01858	139	37	Hypothetical protein	-	-	+	-	-	-
01859	539	37	Hypothetical protein	-	-	+	-	-	-
01860	273	37	DNA methyltransferase protein	-	-	+	-	-	-

[A]PTS = Phosphoenolpyruvate-dependent carbohydrate transport system. [B]TRAP = Tripartite ATP-independent periplasmic transport
